# The Story of the Hospitals

**Published:** 1887-07-16

**Authors:** 


### The Story of the Hospitals.

(*Continued from p. 124, vol. ii.*)

### Guy's Hospital.

Guy's Founded. This great institution, which hides its extensive proportions in a by-street in the Borough, and which is hemmed in on all sides by railway stations, warehouses, and hundreds of small and thickly inhabited houses, is the oldest hospital establishment, apart from such monastic foundations as St. Bartholomew's and St. Thomas's, in London. The Westminster Hospital, which, like Guy's, is a purely secular charity, commenced operations in one or two houses, rudely adapted to the purpose, some two years or so before; but the subject of this article was the first hospital built and equipped out of non-ecclesiastical revenues. The founder, Thomas Guy, who flourished as a stationer in the City from 1668 to 1724, was a Governor and a liberal supporter of St. Thomas's Hospital, to whom the land on which Guy's was built originally belonged; and he lived just long enough to see the latter finished. Had he survived another week he would have had the satisfaction of knowing that its beneficent work had actually commenced. The building and the endowments necessary for the maintenance of four hundred patients cost him two hundred and fifty thousand pounds, and since the opening of the hospital until 1871 the premises have been continually enlarged; and now there is accommodation for 650 beds, in addition to museum, class-rooms, and every appliance necessary for the carrying on of a great medical school. Unfortunately, however, the operations of the hospital have been very much restricted within recent years, for agricultural depression has told with terrible effect upon the rent-roll of the hospital property, and various are the devices which have to be resorted to to make both ends meet, even with the diminished scale on which the hospital work is now conducted. It is worthy of note that, prior to the year 1879—thanks to Guy's beneficence, and a legacy amounting to £180,000 acquired in 1828, under the will of William Hunt of Petersham—the Governors were enabled to carry on their great work for the healing of the sick and the relief of the suffering for over a hundred and fifty years without a single appeal to the charitable public. *Tempora mutantur!* Now they are only too glad to share in the various funds which modern philanthropy has inaugurated for the benefit of this and similar charities.

Inside the Hospital. On entering the hospital by the main gateway in St. Thomas's Street the attention of the visitor is arrested by an ancient and somewhat dilapidated statue of the founder. On the left is the east wing, containing the treasurer's house, the governor's court-room and offices, the superintendent's residence, and the house-surgeons' and dressers' rooms; on the right is the west wing, which includes the chapel and the residences of the chaplain and the matron. The main building is the original Guy's Hospital. A broad colonnade runs through it, and on the right and left are flagged courtyards where, on a fine morning, those inmates of the accident wards who are able to wheel themselves about in invalid-chairs may be seen grouped together, smoking or discussing the contents of the day's paper. One or both of the nether limbs of these men are swathed in bandages, and some of their faces are drawn with suffering, but their cheerful conversation and ready greetings betray contentment, and one can fancy that, were it not for the knowledge that wife and children are temporarily deprived of the bread-winner, they will be very sorry to leave these friendly walls. As originally designed, there was a broad corridor opening out of each side of the colonnade which formed an indoor promenade extending right round the building, but these have long since been converted into the accident wards, and extremely light, airy, comfortable wards they look. It was surprising to be told that no less than fifty accidents per day—taking the average of the year—come to be treated at Guy's Hospital, and of these about ten per cent. are detained, the remainder having their wounds dressed and being then sent home. With so many claims on their resources, the staff are compelled to refuse admittance in any case in which the patient is able to walk to his or her home; but even with this hard-and-fast rule most of the beds were occupied on the occasion of our visit. Fractures of the limbs or the skull in nearly every case were the injuries; and one could not help being impressed with the conviction that in very few well-to-do homes could such appliances for relief and treatment be provided as were here in abundance. The majority of the accidents received in these wards have been met with in the workshops and railway sidings of the locality. Close by are the dressers' rooms, where urgent cases, not necessarily accidents, are received. Here one is always sure of seeing a motley throng, and a glance through the book of daily applicants for medical or surgical attendance revealed to us the surprising fact that four-fifths of the visitors come to be treated for diarrhœa (it was in early September that our visit was paid), and having obtained a draught they go away rejoicing—or, at any rate, relieved. A nominal charge is made here, as in the out-patients' department, when the applicants can afford it; but of this we shall have something to say later on.

The rest of the wards in this old building are occupied only by surgical cases;—

About the Wards.

and here we may pause to remark that all the wards in the hospital have Biblical — generally Old Testament— names. Ruth, Barnabas, Miriam, John, Cornelius, are to our minds more fitting designations than those which are so often given to commemorate a founder or to recall the existence of some eminent person at the time the ward was opened. The cases in the surgical wards on the occasion of our visit were, of course, numerous, but none of them presented any striking features. One lady, whose plump and healthy face suggested a dairy farm rather than a London hospital, was being measured for a pair of French crutches, for which the Salvation Army was to pay. The patient had attained to the dignity of staff captain in that militant body of Christians, and was looking forward eagerly to resuming her work in the cause of religion. She was evidently a favourite with nurses and patients alike. There were a good many children on both the male and female sides, boys over seven years of age being placed with the men, and these little people looked happier than anybody else. In fact, they not unfrequently display a patience under suffering which might teach some of their elders a lesson; and we could not but marvel at the contentment which shone upon the faces of two or three children, although the spinal complaints from which they were suffering necessitated them being retained, by means of a metal frame fastened to the bed, in one position. The cots for the very little children have collapsable sides, so that the tiny inmates may be readily got at. As for the ordinary beds in all the wards, they are made upon the steel spring mattresses which are now being so universally adopted in hospitals; but for cases of fracture, where a firm basis is needed, the steel gives place to strong wooden laths. In all the wards at Guy's there is an ingenious contrivance, designed by Dr. Steele, the medical superintendent, for utilising the coal-box, and for preventing the noise and clatter which the use of ordinary scuttles would entail. The box itself is as long as one of the beds, and is mounted upon wheels fitted with india-rubber tyres. The top of the box is fitted with mattress, pillows, etc., so as to form a very comfortable couch. At one end of the box is an iron shutter and a guard. The former being raised, the coal falls into the latter. As the supply of fuel which this receptacle is capable of containing is very considerable, the saving of dirt, noise, and trouble is considerable. The invalid travelling chairs in the hospital are also designed by the superintendent. They have the advantage of being fifty per cent. cheaper and quite as effective as ordinary chairs—a very important consideration when funds are low.

Above these wards is what is called in the hospital, somewhat contemptuously, the Garret, because of its sloping roof—a light, airy, and very pleasantly situated apartment.

In the "Garret."

From its old-fashioned windows some historical hostelries are to be seen,—the White Hart, where Jack Cade's short-lived triumph came to an ignominious end by the desertion of his Kentish followers; the George, where Mr. Pickwick first encountered Sam Weller; and the Tabard, whence Chaucer's Canterbury Pilgrims started on their memorable journey. This "garret" was originally intended to be used as an isolation ward for patients from other parts of the hospital who were suffering from erysipelas; but as there are no cases of the sort in the institution, some of the beds are occupied by ordinary patients. A former Sister of this ward has decorated a screen with representations of medicinal plants—the opium poppy, the hellebore, the male shield fern, and others. Of hand-painted screens, by the way, there is a great variety at Guy's, many ladies having utilised their leisure and their art in this thoughtful fashion, whilst others have similarly decorated the Sisters' rooms and even the walls of some of the wards.

Returning to the colonnade and passing thence into the grounds—a veritable oasis in the midst of a brick and mortar desert—one sees on the right the new building, or Hunt's House, completed in 1850; and standing by itself in the middle of a grass-plot, a little one-storey building.

A bit of Old Guy's.

This dates from 1774, and was erected for the accommodation of twenty confirmed lunatics, in accordance with the terms of Guy's will. Less than thirty years ago the cells, arranged on what we should now call the inhuman principles adopted a hundred years back, were inhabited by incurable lunatics, though the iron chains and other ancient appliances were, of course, never used. In 1859 the lunatics were provided for elsewhere, the cells were demolished, five large windows were inserted in the walls, and now, on the right and left of the entrance-way are two bright, airy wards devoted to clinical cases—cases, that is, presenting special features which necessitate more constant observation and study than those in the other wards. One has to be told this, however, or the appearance of the inmates would not lead to any such supposition. A good many of the patients were children, who were amusing themselves with a quiet earnestness, contrasting very forcibly with the noisy rompings of children of the same age out of doors.

(*To be continued.*)

